# Vitamin D Insufficiency May Account for Almost Nine of Ten COVID-19 Deaths: Time to Act. Comment on: “Vitamin D Deficiency and Outcome of COVID-19 Patients”. *Nutrients* 2020, *12*, 2757

**DOI:** 10.3390/nu12123642

**Published:** 2020-11-27

**Authors:** Hermann Brenner, Ben Schöttker

**Affiliations:** 1 Division of Clinical Epidemiology and Aging Research, German Cancer Research Center, 69120 Heidelberg, Germany; b.schoettker@dkfz.de; 2 Network Aging Research, University of Heidelberg, 69120 Heidelberg, Germany

Evidence from observational studies is accumulating, suggesting that the majority of deaths due to SARS-CoV-2 infections are statistically attributable to vitamin D insufficiency and could potentially be prevented by vitamin D supplementation. Given the dynamics of the COVID-19 pandemic, rational vitamin D supplementation whose safety has been proven in an extensive body of research should be promoted and initiated to limit the toll of the pandemic even before the final proof of efficacy in preventing COVID-19 deaths by randomized trials.

We read, with great interest, the recent article by Radujkovic et al. that reported associations between vitamin D deficiency (25(OH)D < 12 ng/mL) or insufficiency (25(OH)D < 20 ng/mL) and death in a cohort of 185 consecutive symptomatic SARS-CoV-2-positive patients admitted to the Medical University Hospital Heidelberg, who were diagnosed and treated between 18 March and 18 June 2020 [1]. In this cohort, 118 patients (64%) had vitamin D insufficiency at recruitment (including 41 patients with vitamin D deficiency), and 16 patients died of the infection. With a covariate-adjusted relative risk of death of 11.3, mortality was much higher among vitamin D insufficient patients than among other patients. When translated to the proportion of deaths in the population that is statistically attributable to vitamin D insufficiency (“population attributable risk proportion”), a key measure of public health relevance of risk factors [2], these results imply that 87% of COVID-19 deaths may be statistically attributed to vitamin D insufficiency and could potentially be avoided by eliminating vitamin D insufficiency.

Although results of an observational study, such as this one, need to be interpreted with caution, as done by the authors [1], due to the potential of residual confounding or reverse causality (i.e., vitamin D insufficiency resulting from poor health status at baseline rather than vice versa), it appears extremely unlikely that such a strong association in this prospective cohort study could be explained this way, in particular as the authors had adjusted for age, sex and comorbidity as potential confounders in their multivariate analysis. There are also multiple plausible mechanisms that may well explain the observed associations, such as increased concentrations of pro-inflammatory cytokines, as well as decreased concentrations of anti-inflammatory cytokines in vitamin D insufficiency [3,4]. Although final proof of causality and prevention of deaths by vitamin D supplementation would have to come from randomized trials which meanwhile have been initiated (e.g., [5]), the results of such trials will not be available in the short run. Given the dynamics of the COVID-19 pandemic and the proven safety of vitamin D supplementation, it therefore appears highly debatable and potentially even unethical to await results of such trials before public health action is taken. Besides other population-wide measures of prevention, widespread vitamin D_3_ supplementation at least for high-risk groups, such as older adults or people with relevant comorbidity, which has been proven by randomized controlled trials to be beneficial with respect to prevention of other acute respiratory infections and acute acerbation of asthma and chronic pulmonary disease [6,7,8,9,10], should be promoted. In addition, targeted vitamin D_3_ supplementation of people tested SARS-CoV-2-positive may be warranted.
